# Sensitivity to an Illusion of Sound Location in Human Auditory Cortex

**DOI:** 10.3389/fnsys.2017.00035

**Published:** 2017-05-23

**Authors:** Nathan C. Higgins, Susan A. McLaughlin, Sandra Da Costa, G. Christopher Stecker

**Affiliations:** ^1^Department of Hearing and Speech Sciences, Vanderbilt University School of MedicineNashville, TN, United States; ^2^Institute for Learning and Brain Sciences, University of WashingtonSeattle, WA, United States; ^3^Biomedical Imaging Research Center (CIBM), School of Basic Sciences, Ecole Polytechnique Fédérale de LausanneLausanne, Switzerland

**Keywords:** auditory cortex (AC), binaural hearing, spatial localization, fMRI neuroimaging, auditory perception, auditory illusion

## Abstract

Human listeners place greater weight on the beginning of a sound compared to the middle or end when determining sound location, creating an auditory illusion known as the Franssen effect. Here, we exploited that effect to test whether human auditory cortex (AC) represents the physical vs. perceived spatial features of a sound. We used functional magnetic resonance imaging (fMRI) to measure AC responses to sounds that varied in perceived location due to interaural level differences (ILD) applied to sound onsets or to the full sound duration. Analysis of hemodynamic responses in AC revealed sensitivity to ILD in both full-cue (veridical) and onset-only (illusory) lateralized stimuli. Classification analysis revealed regional differences in the sensitivity to onset-only ILDs, where better classification was observed in posterior compared to primary AC. That is, restricting the ILD to sound onset—which alters the physical but not the perceptual nature of the spatial cue—did not eliminate cortical sensitivity to that cue. These results suggest that perceptual representations of auditory space emerge or are refined in higher-order AC regions, supporting the stable perception of auditory space in noisy or reverberant environments and forming the basis of illusions such as the Franssen effect.

## Introduction

**Figure 1:**
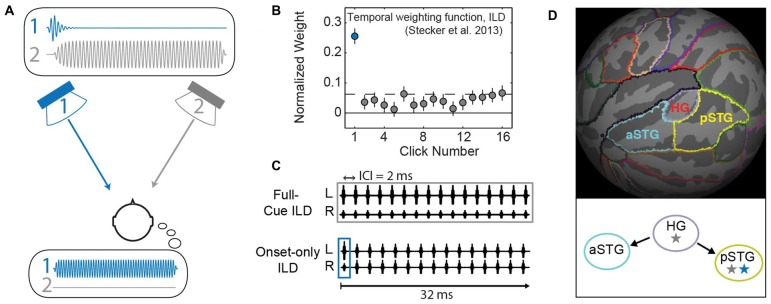
**Background and methods. (A)** In the Franssen effect, a tone is briefly presented from one loudspeaker (1, blue), and quickly cross-faded to a second loudspeaker (2, gray). Although only loudspeaker 2 sounds during most of the tone’s duration (upper inset plots physical waveform), listeners erroneously perceive the sound to emanate from the loudspeaker that presented the onset (i.e., speaker 1; lower inset illustrates the resulting percept). This type of effect can be quantified by measuring a “temporal weighting function” that plots the perceptual “weight” which listeners apply to each part of the sound duration (Stecker et al., 2013). **(B)** The temporal weighting function for interaural level differences (ILD) carried by a train of 16 clicks repeating at 2 ms ICI reveals largest weight on the first click (blue), consistent with onset dominance as seen in the Franssen effect. **(C)** Click-train stimuli modified from Stecker et al. (2013) were used in the current study. Upper: in the full-cue condition, all 16 clicks carry the same ILD value. Lower: the onset-only condition tests ILD carried only by the first click; other clicks carry zero ILD, thus providing a simulation of the Franssen effect over headphones. **(D)** Regions of interest (ROIs) were defined on the average cortical surface (FSaverage). Colors indicate boundaries of Heschl’s gyrus (HG), anterior superior temporal gyrus (aSTG) and posterior superior temporal gyrus (pSTG) relative to cortical gyri (light gray) and sulci (dark gray). Lower panel illustrates “dual-stream” theories of primate auditory cortex (AC; Rauschecker and Scott, 2009): AC core regions (HG) exhibit sensitivity to stimulus features including binaural cues (gray star). Information propagates from core to anterior regions (aSTG) that lack spatial sensitivity (no star) and to posterior regions (pSTG) that maintain sensitivity to binaural/spatial cues (gray star) and are implicated in the perceptual processing of auditory space (blue star).

When human listeners localize sounds in space they make use of several different acoustic cues, including interaural time differences (ITD) and interaural level differences (ILD), as well as monaural spectral cues. In real world listening, the magnitude and reliability of each cue varies significantly across frequency and over time; however, the perception of sound location remains stable. That is, not all cues affect perception with equal weight. In particular, binaural cues present at sound onset tend to dominate perception (Stecker et al., 2013), a phenomenon that is dramatically illustrated by the Franssen effect (Franssen, 1960; Hartmann and Rakerd, 1989), and depicted in Figure 1A. This illusion occurs when the onset (i.e., the first few milliseconds) and the remainder of a sound are presented from different loudspeakers in a room. The entirety of the sound is perceived to emanate from the onset loudspeaker for several seconds or more, even though the other loudspeaker presents nearly all of the sound energy. The illusion of the Franssen effect thus illustrates “onset dominance” in sound localization and reveals a powerful dissociation between the spatial perception and the physical features of an auditory stimulus. The Franssen effect is strong for tonal stimuli presented in reverberant space, a situation in which ongoing cues are rendered ambiguous. Onset dominance in periodic headphone-presented sound shares many characteristics with the Franssen illusion (Stecker et al., 2013), rendering these aspects open to investigation in controlled environments and by techniques such as functional magnetic resonance imaging (fMRI).

Several human imaging studies have exploited optical illusions in order to distinguish physical vs. perceptual representations in the visual cortex. For example, Murray et al. (2006) showed that blood oxygenation level dependent (BOLD) activity in primary visual cortex (V1) scales with the perceived size of an object and not simply the extent of its retinal image. Similarly, Barendregt et al. (2015) used binocular disparity to reveal a transformation from representations of retinal position in human V1 to perceived cyclopean (unified binocular) position in V2. Thus, for different dimensions of visual stimuli, Murray et al. (2006) demonstrated perceptual representations in V1, whereas Barendregt et al. (2015) demonstrated the emergence of perceptual representations across cortical regions. Single-unit recording methods have been similarly employed, and Rajala et al. (2013) recently demonstrated strong correlations between neural activity in the rhesus monkey inferior colliculus and behavioral responses to Franssen stimuli, suggesting a possible subcortical origin of the illusion. The current study addresses whether similar perceptual—as opposed to physical representations—are at work in the human auditory cortex (AC), and if they emerge across the hierarchy of cortical regions (Yagcioglu and Ungan, 2006).

Sound-driven responses in the AC are broadly tuned to the spatial features of sounds, particularly the ILD. Typically, stronger responses are elicited by sounds that are more intense in the contralateral ear (corresponding to sound sources located in the contralateral hemifield), as measured via single unit recordings in animal models (Phillips and Irvine, 1981; Lui et al., 2015) and by BOLD fMRI in human listeners (Jäncke et al., 2002; Stecker et al., 2015; McLaughlin et al., 2016). It is not known, however, whether this contralateral dominance reflects a *sensory* representation of the physical cues or a *perceptual* representation of contralateral auditory space.

In order to investigate whether spatial tuning in the human AC reflects the perceived or the physical features of sounds, we used fMRI to measure BOLD-response tuning to ILD carried by rapid trains of high-frequency narrowband clicks. Here, ILD cues were applied to the initial click in each train, and BOLD responses compared to a companion data set (McLaughlin et al., 2016) that applied ILD to all clicks in each train. Previous work in our lab has quantified listeners’ Franssen-like perception of such sounds, in that spatial judgments are strongly dominated by the binaural features of the first (onset) click (Figure 1B) even when other clicks are present with different cue values (Brown and Stecker, 2010; Stecker et al., 2013). Importantly, these psychoacoustical results validate onset-dominated spatial perception using headphone stimulation compatible with fMRI studies, and informed the choice of stimuli used in this study. If AC responses reflect the behaviorally resolved *spatial perception*, similar ILD tuning should be observed regardless of whether the cue is applied to all clicks or only to the first, since the remaining clicks are illusorily perceived at the initial location. On the other hand, if AC responses reflect the *physical spatial cues*, they should reveal minimal to non-existent tuning to the onset-only ILD stimuli, since the majority of clicks are presented with a fixed 0 dB ILD.

## Materials and Methods

Data from nine participants (five female) were collected at the University of Washington Diagnostic Imaging Sciences Center in Seattle, WA, USA. The full-cue and onset-only ILD datasets were collected in the same imaging session for all participants. The full-cue dataset was described in a previous publication (McLaughlin et al., 2016) using alternate analytical approaches. Here, those data were reanalyzed and comprise the comparison, or control dataset (“full-cue” condition). The experimental protocol, imaging and preprocessing analysis were identical to those described in that study. All participants were between 18 years and 35 years old, right handed, with no tonal language experience, no known history of neurological disorders, and no contra-indications to MR scanning. Pure-tone audiometry confirmed normal hearing in all participants (thresholds within 20 dB of normal in each ear at all octave frequencies from 500 Hz to 8000 Hz). This study was carried out in accordance with the recommendations and guidelines of the University of Washington Human Subjects Division with written informed consent from all subjects. All subjects gave written informed consent in accordance with the Declaration of Helsinki. The protocol was approved by the University of Washington Institutional Review Board.

*Stimuli* were presented in trials of 1-s duration. Within each trial, four brief (151-ms) stimuli were presented in successive randomly-timed intervals. The individual stimuli were trains of 16 Gaussian-filtered impulses (“Gabor clicks”) presented at a click rate of 500 per second (one click per 2 ms). Stimulus center frequency was 4000 Hz and −3 dB bandwidth was 0.8 octaves (2230 Hz). The use of multiple intervals per trial subserved the task element of the experiment, in which participants detected occasional pitch deviation in one of the intervals (i.e., the task was four-interval, same/different discrimination). The ILD value for each trial was assigned (identically on all four intervals) pseudorandomly from a set of nine ILD values (±30, ±20, ±10, ±5, 0 dB). By convention, negative values indicate leftward ILD (greater intensity in the left ear). ILD was implemented by increasing level by half the test ILD value in one ear and decreasing level by half the test value in the other ear. Except for an equally likely tenth “silent” condition presented at an inaudible level (−10 dB SPL; not included in this analysis), sounds were presented at an average binaural level (ABL) of 80 dB SPL. The inter-trial time ranged randomly from 0 s to 5 s. The order of ILD conditions followed a continuous carryover paradigm (Aguirre, 2007), to ensure equal numbers of trials for each possible pairing of conditions (0 dB followed by +20 dB, etc). Thus, stimulus trials were pseudorandomly counterbalanced across 20 trials per condition (200 trials total) within each imaging run.

In the *full-cue* ILD experiment (McLaughlin et al., 2016), all 16 clicks in each train carried the same ILD value (Figure 1C; upper), which varied from trial to trial. In the *onset-only* condition tested here (Figure 1C; lower), the ILD of the first click (the onset) varied from trial to trial; clicks 2–16 carried zero ILD and did not vary from trial to trial. Sounds were presented using custom Matlab (Mathworks, Natick, MA, USA) routines, synthesized with Tucker Davis Technologies RP2.1 (Alachua, FL, USA), and delivered to listeners via piezoelectric insert earphones (Sensimetrics S14, Malden, MA, USA) enclosed within circumaural ear defenders to attenuate scanner noise by ~40 dB.

*Imaging* was performed at 3 Tesla (Philips Achieva, Eindhoven Netherlands). In both the full-cue (McLaughlin et al., 2016), and onset-only condition each participant completed two imaging runs, approximately 10 min each. A high-resolution T1-weighted whole brain structural image (MPRAGE) was also acquired for each participant and used for registration of functional data and cortical surface extraction using Freesurfer 4.1 (Martinos Center for Biomedical Imaging, MGH, Boston, MA). Functional scans acquired BOLD data using a continuous event-related imaging paradigm (echo-planar imaging, TR = 2 s, 42 slices, 2.75 × 2.75 × 3 mm resolution). Subjects were instructed to fixate on a visual center cross projected onto a visible screen, and to indicate (by button press) when infrequent pitch-change “targets” occurred. Targets occurred randomly, on average once per 13 s, and consisted of increased click rate on the third interval of a trial, perceived as a change in pitch. Note that the task paradigm requires discrimination along a dimension (pitch change) orthogonal to the experimental manipulation of interest (ILD condition). This approach was chosen to ensure active listening on the part of the participant without introducing response and attention variables. In addition to sound delivery, custom Matlab routines were used to log event timing for stimulus presentation, target presentation, responses and scanner acquisition triggers.

### Preprocessing and ROI Analyses

Functional data for each run were pre-processed using FEAT (FSL 4.1, FMRIB, Oxford, UK (Smith et al., 2004)) to perform high-pass filtering (100 s), motion correction, B0 unwarping and skull-stripping. In each hemisphere, AC was parcellated into three regions of interest (ROIs): Heschl’s gyrus (HG), anterior superior temporal gyrus (aSTG) and posterior superior temporal gyrus (pSTG) using the Freesurfer-provided Desikan-Killiany atlas (Desikan et al., 2006). Using this atlas, the superior temporal gyrus (STG) was subdivided into anterior and posterior STG regions at its intersection with HG (McLaughlin et al., 2016). To create subject-specific ROIs, template ROIs were defined on the Freesurfer average surface, mapped to each individual’s cortical surface, and projected to his/her functional 3-D volume (Figure 1D).

### Functional Univariate Analysis

**Figure 2:**
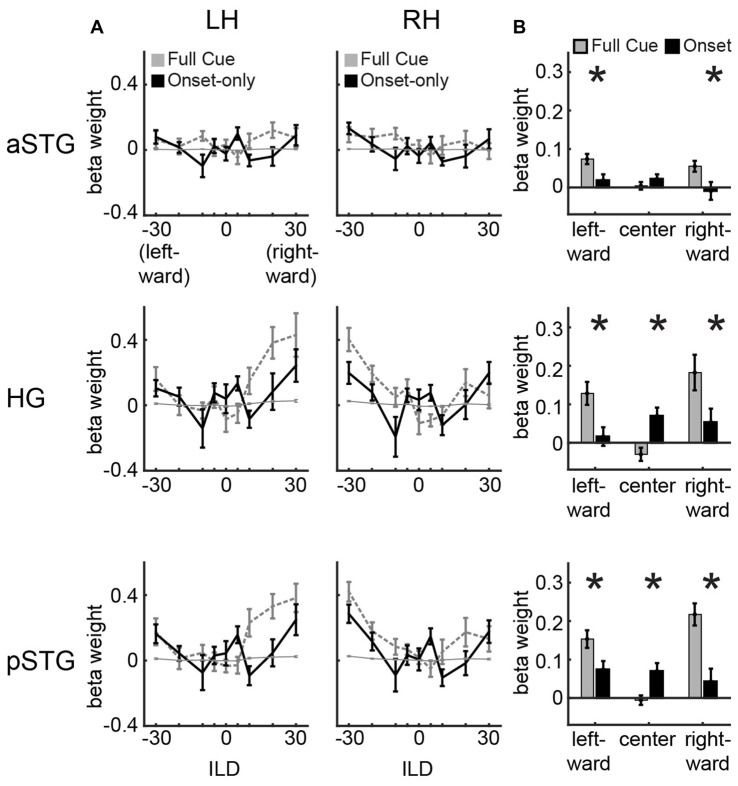
**Univariate results. (A)** Univariate ILD tuning functions plot average beta weights for all voxels in left and right hemisphere for aSTG, HG and pSTG in response to sounds varying in ILD in the full-cue (veridical lateralization; dashed gray lines) and onset-only (illusory lateralization; black lines) conditions. Thin gray line depicts 1/16th of the full-cue tuning function, indicating the hypothetical response to the physical features of the onset-only sound. **(B)** Average of beta weights across left and right hemisphere in response to leftward (−30, −20, −10 dB ILD), center (−5, 0, +5 dB ILD) and rightward (+10, +20, +30 dB ILD) sounds for the full-cue (gray) and onset-only (black) stimuli. Error bars reflect SEM across participants. Asterisks reflect significant difference based on paired *t*-test (*p* < 0.05; FDR controlled).

Trial-to-trial measures of activation were quantified by extracting and temporally interpolating the 12-s signal time course aligned to the onset of each trial, followed by linear regression of the interpolated time course with a hemodynamic response function adapted from Glover (1999), using FMRISTAT Matlab toolbox^1^. The function was defined by the difference of two gamma functions with the following parameters: 3.5 (Peak 1), 5.2 (FWHM 1), 10.8 (Peak 2), 7.35 (FWHM 2), 0.35 (Dip), where 1 and 2 refer to the first and second gamma functions. The resulting beta weight defined the response magnitude at each voxel on each trial. Univariate ILD tuning functions (Figure 2) were quantified by averaging across voxels in each ROI and calculating the main effect of ILD modulation using repeated-measures ANOVA for each hemisphere for each region. Multi-voxel classification (see below) made use of the trial- and voxel-specific responses without averaging.

### Multi-Voxel Pattern Analysis

**Figure 3:**
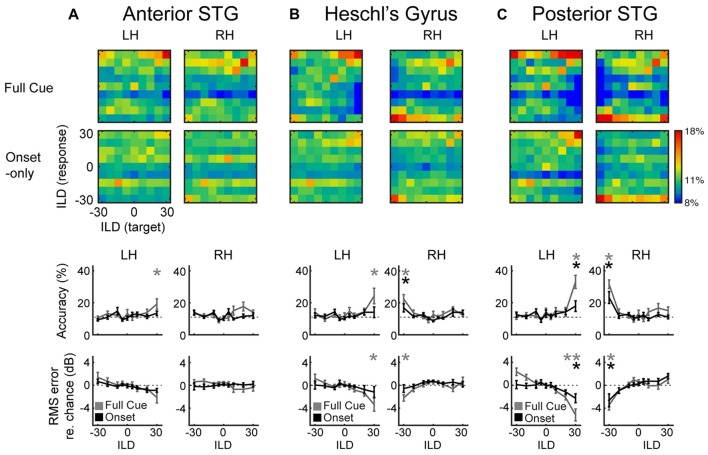
**Multi-Voxel Pattern Analysis (MVPA) results for (A)** aSTG, **(B)** HG and **(C)** pSTG, show confusion matrices for the full-cue (first row) and onset-only (second row) stimuli. Color scale indicates classification rate. Target (presented) ILDs are organized along the *x*-axis, and response (predicted) ILDs along the *y*-axis. Third and fourth rows plot mean accuracy and root-mean squared (RMS) error with respect to chance, as a function of ILD for full-cue (gray) and onset-only (black) conditions. Dashed lines indicate level of chance performance (11% accuracy or 0% RMS error); asterisks indicate significant difference with respect to the permuted dataset (*p* < 0.05).

Patterns of voxel activity were extracted for three ROIs (HG, aSTG and pSTG). Separately for each ROI, half of the trials were selected randomly and used to train a linear classifier (LIBSVM; Chang and Lin, 2011) on the nine ILD conditions. The remaining trials formed a test set for independent cross-validation of classification performance. Classification data were saved into a confusion matrix, and the entire process (selection of training and test sets and cross-validation) was repeated 1000 times for each ROI in each hemisphere. A separate permutation dataset was similarly generated by randomly shuffling the ILD trial labels in order to estimate the sampling distribution of classification performance and allow permutation tests of significance. Statistical analyses of classification results were conducted using this 1000-fold permutation test, and reported to one significant digit. Classification accuracy was defined as the probability of correct classification, and root-mean squared error measurements (RMS error) were calculated to quantify the magnitude (in units of dB ILD) of errors observed for each condition. RMS error data in Figure 3 are presented relative to chance performance to allow clearer visualization of departures from 0.

## Results

### Task Performance

Behavioral data for the pitch detection task was collected from the nine participants during the four imaging runs. Performance was quite good (mean *d’* = 2.4, *σ* = 0.48), indicating that participants actively listened to the sound stimuli. No performance differences were observed between runs or stimulus type. Participants were not asked to make speeded responses; nevertheless, response times were consistent across subjects with a mean of 1.4 s (*σ* = 0.12 s) calculated from the onset of each sound trial.

### Univariate Analyses Reveal Bilateral Tuning to Full-Cue and Onset-Only ILD in HG and pSTG

Figure 2A plots ILD tuning functions in each ROI (rows) and hemisphere (columns). Within each panel, dashed gray lines plot full-cue ILD tuning functions extracted from ILD data of McLaughlin et al. (2016). These show no modulation to ILD in aSTG (right aSTG: *F*_(8,8)_ = 0.76, n.s.; left aSTG: *F*_(8,8)_ = 0.72, n.s.) but significant modulation in HG and pSTG (right HG: *F*_(8,8)_ = 5.63, *p* < 0.05; left HG: *F*_(8,8)_ = 4.45, *p* < 0.05; right pSTG: *F*_(8,8)_ = 3.28, *p* < 0.05; left pSTG: *F*_(8,8)_ = 4.28, *p* < 0.05). These data are also re-plotted after scaling by a factor of 1/16 (thin gray line) to indicate the “average physical cue” expectation for ILD tuning in the onset condition. Because the majority of clicks in the onset condition carry 0 dB ILD, the hypothetical tuning functions appear quite flat, reflecting the null hypothesis that AC activity follows the physical cues rather than the illusory spatial perception induced by the onset-only stimuli. In contrast to that expectation, ILD tuning functions observed in the onset-only condition (solid black lines) show clear modulation by ILD (Figure 2A; right HG: *F*_(8,8)_ = 3.0, *p* < 0.05; left HG: *F*_(8,8)_ = 1.86, n.s.; right pSTG: *F*_(8,8)_ = 3.01, *p* < 0.05; left pSTG: *F*_(8,8)_ = 2.17, *p* < 0.05), which appears generally consistent with the shape of full-cue ILD tuning functions. HG and pSTG demonstrate greater activity at contralateral and midline, relative to intermediate, ILD values. aSTG demonstrates minimal modulation to onset ILD cues (Figure 2A; right aSTG: *F*_(8,8)_ = 1.60, n.s.; left aSTG: *F*_(8,8)_ = 1.96, n.s.), as expected from lack of modulation to full-cue ILDs. Comparison of activation across full-cue and onset-only conditions for leftward (−30, −20, −10 dB ILD) and rightward (+10, +20, +30 dB ILD) conditions reveals significantly larger responses to the full-cue stimuli (Figure 2B, asterisks denote significance based on paired *t*-test, all controlled for false discovery rate; minimum *t-value* reported for leftward *t*_(8)_ = 2.6, *p* < 0.05; rightward *t*_(8)_ = 2.23, *p* < 0.05). An additional (and unexpected) feature of tuning to onset ILD is the enhanced response to near-midline (−5, 0, 5 dB) positions in the onset-only vs. full-cue condition, visible as a bump in the tuning function in all ROIs (black line), and statistically greater for the onset in HG (*t*_(8)_ = 4.2, *p* < 0.05) and pSTG (*t*_(8)_ = 2.8, *p* < 0.05).

### Multi-Voxel Analyses Reveal Robust Classification of Full-Cue (Physical) ILD in HG and pSTG, and of Onset-Only (Perceived) ILD in pSTG

The sensitivity of AC responses to ILD is clearly apparent in the tuning functions of Figure 2A. Yet some forms of sensitivity can be masked in the overall response. For example, some neural populations may increase their response while others decrease their response with changes in ILD. Multi-voxel patterns can sometimes capture such differences. Successful classification of patterns provides broader evidence of ILD sensitivity than do average tuning functions alone. Figure 3 plots the results of such an analysis. Trial-to-trial voxel patterns in each ROI were used to train and cross-validate ILD classification by a support vector machine. To our knowledge, this is the first auditory demonstration of multi-voxel scaling along a parametrically manipulated stimulus dimension, as opposed to binary classification of discrete categories. Successful classification of ILD values was observed across a range of conditions, with performance exceeding chance levels by up to 22%. That performance is roughly on par with other attempts at multi-voxel classification in the auditory system (De Martino et al., 2008; Obleser et al., 2010; Kumar et al., 2014; Gardumi et al., 2016).

Confusion matrices representing classification percentage reveal a limited ability of aSTG patterns to classify ILD in either condition (Figure 3A). Classification accuracy in aSTG was significantly above chance, as determined by 1000-fold permutation test (*p* < 0.02) only in the left hemisphere for the full-cue +30 dB (contralateral) condition (Figure 3A; third row). In HG (Figure 3B), significant classification of full-cue ILD was found in both right (accuracy: *p* < 0.001; RMS error: *p* < 0.03) and left (accuracy: *p* < 0.001; RMS error: *p* < 0.003) hemispheres for the most contralateral values. ILD classification by HG in the onset-only condition was poor, and above chance only for the −30 dB ILD condition in right HG (Figure 3B; accuracy: *p* < 0.05, RMS error: *p* < 0.05). The best classification performance was observed in pSTG (Figure 3C): contralateral full-cue ILDs were successfully classified for the most contralateral ILDs in right (accuracy: *p* < 0.001; RMS error: *p* < 0.002) and left hemispheres (accuracy: *p* < 0.001; RMS error: *p* < 0.001), as were contralateral onset-only ILDs (Right hemisphere accuracy: *p* < 0.001, RMS error: *p* < 0.02; Left hemisphere accuracy: *p* < 0.01, RMS error: *p* < 0.03). In the onset-only condition, direct comparison of ILD classification accuracy across regions of right AC reveals significantly higher accuracy in pSTG than HG (paired *t*-test: *t*_(8)_ = 4.3, *p* < 0.05).

## Discussion

The results of this study suggest that BOLD responses in human AC are sensitive to the perceived, and not merely the physical features of sound location. In particular, restricting the ILD to the onset of a high-frequency sound does not eliminate tuning to that cue in BOLD responses, just as it does not eliminate the lateral perception of such sounds (Figure 1B). Notably, the greatest ILD sensitivity—in both full-cue and onset-only conditions—was observed in core and posterior regions of the AC (HG and pSTG; Figure 1D). That observation is consistent with previous studies of human AC (Yagcioglu and Ungan, 2006; Stecker et al., 2015; McLaughlin et al., 2016), and with the proposed specialization of posterior AC regions, as part of a dorsal “stream,” for spatial processing and perception (Maeder et al., 2001; Tian et al., 2001; Warren and Griffiths, 2003; Lomber and Malhotra, 2008).

In both HG and pSTG, BOLD response tuning to onset-only ILD remained significantly more modulated than expected for simple tuning to the average cue physically present in the stimulus. The magnitude of ILD modulation was reduced, however, when compared to the full-cue condition (Figure 2), suggesting that both the onset and the later portions of the sound contributed to ILD tuning. That result is in fact quite consistent with psychophysical studies using similar stimuli (Stecker and Hafter, 2009; Stecker and Brown, 2010; Stecker et al., 2013). Those studies have repeatedly demonstrated that although the first click’s ILD has the largest overall impact on sound localization, the ILD of the last few clicks also contributes significantly. Equal weighting of the initial and final ILD would result in approximately 50% reduction in response modulation by ILD, a value that appears roughly consistent with the tuning functions of Figure 2A. Thus, ILD tuning functions strongly suggest that responses in the human AC (particularly in HG and pSTG) correlate to the perceptually-weighted, not raw, binaural cues and thus generally to *spatial perception*—whether veridical or illusory—rather than the physical stimulus.

It is worth noting that as compared to ILD, the psychophysical weighting of interaural *time* differences (ITD) is more absolutely dominated by the onset ITD cue (see Stecker et al., 2013). Attempts to measure onset-ITD tuning using fMRI have not been successful, however, in part because BOLD response tuning to full-cue ITD appears quite weak (McLaughlin et al., 2016). Future studies should continue to investigate these issues, as they suggest important differences in AC sensitivity to ITD and ILD. Because ITD is widely viewed as the dominant sound-localization cue for human listeners, understanding such differences is critical to relating AC BOLD measurements to sound localization.

Contemporary models of AC organization hypothesize that information flows from core AC regions (e.g., primary AC) to higher-order AC regions located anterior and posterior to the core. Functionally, posterior regions appear more sensitive to the spatial aspects of sounds and tasks, while anterior regions appear more strongly involved with non-spatial aspects such as sound identity (Rauschecker and Scott, 2009). As illustrated in Figure 1D, these hierarchical models suggest the possibility that a perceptual representation of auditory space, subject to spatial illusions, will emerge as a consequence of this information flow from core regions near HG to posterior regions of the superior temporal gyrus (pSTG). The data presented here are at least roughly consistent with that possibility. Multi-voxel pattern analyses (MVPA) revealed greater onset-ILD sensitivity in right pSTG than HG, compared to more equivalent representations of full-cue ILD in both regions. Together with the results of studies in other domains (e.g., categorical speech representation), and with respect to subcortical neural encoding of the Franssen illusion (Rajala et al., 2013) this result supports a cortical hierarchical transformation from relatively more *sensory* representations in HG to more *perceptual* representations in pSTG (Griffiths and Warren, 2004; Rauschecker and Scott, 2009; Chang et al., 2010; Steinschneider et al., 2014). These higher-order perceptual representations exhibit sensitivity to spatial illusions, such as the Franssen effect, and could play a role in emphasizing reliable spatial features (such as sound onsets) that subserve stable perception in noisy and reverberant environments. The results suggest that aSTG does not play a major role in such processes underlying *spatial* perception. Yet it could be that anterior belt regions similarly contribute to stable perception of non-spatial features, such as pattern discrimination (Lomber and Malhotra, 2008; Norman-Haignere et al., 2013).

A surprising feature of the onset-only ILD tuning functions plotted in Figure 2A is the conspicuous “bump” in response to near-midline ILD (±5 dB). Full-cue ILD tuning functions exhibit clear response minima near 0 dB ILD, consistent with previous studies (Stecker et al., 2015). Functions for onset-only ILD, in contrast, exhibit two minima around ±10 dB, and a local maximum in between. This feature is apparent to varying extent in both hemispheres and all ROIs, including anterior STG. We suspect that this feature relates to the 0-dB ILD of the post-onset clicks. As noted above, perceptual weighting for ILD emphasizes both onset and offset clicks (Stecker and Brown, 2012). When the ILD of the onset and offset differ sufficiently, the auditory percept may appear to broaden or move. Note that the short duration (32-ms) of stimuli used here would have minimized the perception of such effects; moreover, participants did not report these effects. Nevertheless, the cortical representations of sounds with similar onset and offset ILD values might reflect greater perceptual fusion of the two values than when onset and offset differ. Previous fMRI literature has, in fact, suggested stronger responses to fused vs. segregated sound features (Huang et al., 2011). It is possible that the ILD tuning bump near 0 dB reflects a similar phenomenon, and thus might relate more strongly to perceptual grouping of onset and offset than to binaural tuning *per se*. It is worth noting that this feature was also evident in aSTG, although attenuated and not statistically significant. That is, despite otherwise minimal sensitivity to ILD, there was a trend for greater response to ILD values around the midline. It could be that anterior STG contains a subset of spatially sensitive voxels that are diluted by a majority of non-spatially sensitive voxels, in this case the bump is just an attenuated version of that observed in HG and posterior STG. More generally, it is not clear whether the position of this bump reflects the similarity in ILD of onset- and post-onset clicks, or potentially a special feature of the region of auditory space corresponding to the auditory midline. Future studies should address this question, for example by opposing onset and offset cues over a broader range of ILD values.

## Author Contributions

SAM and GCS developed and conducted experiments. NCH, SAM, GCS analyzed data. NCH, SAM, SDC and GCS wrote the manuscript.

## Conflict of Interest Statement

The authors declare that the research was conducted in the absence of any commercial or financial relationships that could be construed as a potential conflict of interest.

## Footnotes

^1^http://www.math.mcgill.ca/keith/fmristat/

## References

[B1] Aguirre G. K. (2007). Continuous carry-over designs for fMRI. Neuroimage 35, 1480–1494. 10.1016/j.neuroimage.2007.02.005 17376705 PMC2147064

[B2] Barendregt M. Harvey B. M. Rokers B. Dumoulin S. O. (2015). Transformation from a retinal to a cyclopean representation in human visual cortex. Curr. Biol. 25, 1982–1987. 10.1016/j.cub.2015.06.003 26144967

[B3] Brown A. D. Stecker G. C. (2010). Temporal weighting of interaural time and level differences in high-rate click trains. J. Acoust. Soc. Am. 128, 332–341. 10.1121/1.3436540 20649228 PMC2921433

[B4] Chang C.-C. Lin C.-J. (2011). LIBSVM : a library for support vector machines. ACM Transactions on Intelligent Systems and Technology 2:27, 1–27, 27. Available online at: http://www.csie.ntu.edu.tw/~cjlin/libsvm

[B5] Chang E. F. Rieger J. W. Johnson K. Berger M. S. Barbaro N. M. Knight R. T. (2010). Categorical speech representation in human superior temporal gyrus. Nat. Neurosci. 13, 1428–1432. 10.1038/nn.2641 20890293 PMC2967728

[B6] De Martino F. Valente G. Staeren N. Ashburner J. Goebel R. Formisano E. (2008). Combining multivariate voxel selection and support vector machines for mapping and classification of fMRI spatial patterns. Neuroimage 43, 44–58. 10.1016/j.neuroimage.2008.06.037 18672070

[B7] Desikan R. S. Ségonne F. Fischl B. Quinn B. T. Dickerson B. C. Blacker D.. (2006). An automated labeling system for subdividing the human cerebral cortex on MRI scans into gyral based regions of interest. Neuroimage 31, 968–980. 10.1016/j.neuroimage.2006.01.021 16530430

[B8] Franssen N. V. (1960). Some Considerations On the Mechanisms of Directional Hearing. Delft: Technische Hogeschool.

[B9] Gardumi A. Ivanov D. Hausfeld L. Valente G. Formisano E. Uludag K. (2016). The effect of spatial resolution on decoding accuracy in fMRI multivariate pattern analysis. Neuroimage 132, 32–42. 10.1016/j.neuroimage.2016.02.033 26899782

[B10] Glover G. H. (1999). Deconvolution of impulse response in event-related BOLD fMRI. Neuroimage 9, 416–429. 10.1006/nimg.1998.0419 10191170

[B11] Griffiths T. D. Warren J. D. (2004). What is an auditory object? Nat. Rev. Neurosci. 5, 887–892. 10.1038/nrn1538 15496866

[B12] Hartmann W. M. Rakerd B. (1989). Localization of sound in rooms. IV: the Franssen effect. J. Acoust. Soc. Am. 86, 1366–1373. 10.1121/1.398696 2808910

[B13] Huang Y. Li J. Zou X. Qu T. Wu X. Mao L.. (2011). Perceptual fusion tendency of speech sounds. J. Cogn. Neurosci. 23, 1003–1014. 10.1162/jocn.2010.21470 20350060

[B14] Jäncke L. Wüstenberg T. Schulze K. Heinze H. J. (2002). Asymmetric hemodynamic responses of the human auditory cortex to monaural and binaural stimulation. Hear. Res. 170, 166–178. 10.1016/s0378-5955(02)00488-4 12208550

[B15] Kumar S. Bonnici H. M. Teki S. Agus T. R. Pressnitzer D. Maguire E. A.. (2014). Representations of specific acoustic patterns in the auditory cortex and hippocampus. Proc. Biol. Sci. 281:20141000. 10.1098/rspb.2014.1000 25100695 PMC4132675

[B16] Lomber S. G. Malhotra S. (2008). Double dissociation of ‘what’ and ‘where’ processing in auditory cortex. Nat. Neurosci. 11, 609–616. 10.1038/nn.2108 18408717

[B17] Lui L. L. Mokri Y. Reser D. H. Rosa M. G. P. Rajan R. (2015). Responses of neurons in the marmoset primary auditory cortex to interaural level differences: comparison of pure tones and vocalizations. Front. Neurosci. 9:132. 10.3389/fnins.2015.00132 25941469 PMC4403308

[B18] Maeder P. P. Meuli R. A. Adriani M. Bellmann A. Fornari E. Thiran J. P.. (2001). Distinct pathways involved in sound recognition and localization: a human fMRI study. Neuroimage 14, 802–816. 10.1006/nimg.2001.0888 11554799

[B19] McLaughlin S. A. Higgins N. C. Stecker G. C. (2016). Tuning to binaural cues in human auditory cortex. J. Assoc. Res. Otolaryngol. 17, 37–53. 10.1007/s10162-015-0546-4 26466943 PMC4722015

[B20] Murray S. O. Boyaci H. Kersten D. (2006). The representation of perceived angular size in human primary visual cortex. Nat. Neurosci. 9, 429–434. 10.1038/nn1641 16462737

[B21] Norman-Haignere S. Kanwisher N. McDermott J. H. (2013). Cortical pitch regions in humans respond primarily to resolved harmonics and are located in specific tonotopic regions of anterior auditory cortex. J. Neurosci. 33, 19451–19469. 10.1523/JNEUROSCI.2880-13.2013 24336712 PMC3916670

[B22] Obleser J. Leaver A. M. Vanmeter J. Rauschecker J. P. (2010). Segregation of vowels and consonants in human auditory cortex: evidence for distributed hierarchical organization. Front. Psychol. 1:232. 10.3389/fpsyg.2010.00232 21738513 PMC3125530

[B23] Phillips D. P. Irvine D. R. (1981). Responses of single neurons in physiologically defined area AI of cat cerebral cortex: sensitivity to interaural intensity differences. Hear. Res. 4, 299–307. 10.1016/0378-5955(81)90014-9 7263517

[B24] Rajala A. Z. Yan Y. Dent M. L. Populin L. C. (2013). The inferior colliculus encodes the Franssen auditory spatial illusion. Eur. J. Neurosci. 38, 3056–3070. 10.1111/ejn.12325 23899307 PMC4107190

[B25] Rauschecker J. P. Scott S. K. (2009). Maps and streams in the auditory cortex: nonhuman primates illuminate human speech processing. Nat. Neurosci. 12, 718–724. 10.1038/nn.2331 19471271 PMC2846110

[B26] Smith S. M. Jenkinson M. Woolrich M. W. Beckmann C. F. Behrens T. E. J. Johansen-Berg H.. (2004). Advances in functional and structural MR image analysis and implementation as FSL. Neuroimage 23, S208–S219. 10.1016/j.neuroimage.2004.07.051 15501092

[B27] Stecker G. C. Brown A. D. (2010). Temporal weighting of binaural cues revealed by detection of dynamic interaural differences in high-rate Gabor click trains. J. Acoust. Soc. Am. 127, 3092–3103. 10.1121/1.3377088 21117758 PMC2882667

[B28] Stecker G. C. Brown A. D. (2012). Onset- and offset-specific effects in interaural level difference discrimination. J. Acoust. Soc. Am. 132, 1573–1580. 10.1121/1.4740496 22978886 PMC3460982

[B29] Stecker G. C. Hafter E. R. (2009). A recency effect in sound localization? J. Acoust. Soc. Am. 125, 3914–3924. 10.1121/1.3124776 19507974 PMC2806433

[B31] Stecker G. C. McLaughlin S. A. Higgins N. C. (2015). Monaural and binaural contributions to interaural-level-difference sensitivity in human auditory cortex. Neuroimage 120, 456–466. 10.1016/j.neuroimage.2015.07.007 26163805 PMC4589528

[B30] Stecker G. C. Ostreicher J. D. Brown A. D. (2013). Temporal weighting functions for interaural time and level differences. III. Temporal weighting for lateral position judgments. J. Acoust. Soc. Am. 134, 1242–1252. 10.1121/1.4812857 23927122 PMC3745506

[B32] Steinschneider M. Nourski K. V. Rhone A. E. Kawasaki H. Oya H. Howard M. A. (2014). Differential activation of human core, non-core and auditory-related cortex during speech categorization tasks as revealed by intracranial recordings. Front. Neurosci. 8:240. 10.3389/fnins.2014.00240 25157216 PMC4128221

[B33] Tian B. Reser D. Durham A. Kustov A. Rauschecker J. P. (2001). Functional specialization in rhesus monkey auditory cortex. Science 292, 290–293. 10.1126/science.1058911 11303104

[B34] Warren J. D. Griffiths T. D. (2003). Distinct mechanisms for processing spatial sequences and pitch sequences in the human auditory brain. J. Neurosci. 23, 5799–5804. 12843284 10.1523/JNEUROSCI.23-13-05799.2003 PMC6741275

[B35] Yagcioglu S. Ungan P. (2006). The ‘Franssen’ illusion for short duration tones is preattentive: a study using mismatch negativity. Brain Res. 1106, 164–176. 10.1016/j.brainres.2006.05.075 16831407

